# Digital logic gates in soft, conductive mechanical metamaterials

**DOI:** 10.1038/s41467-021-21920-y

**Published:** 2021-03-12

**Authors:** Charles El Helou, Philip R. Buskohl, Christopher E. Tabor, Ryan L. Harne

**Affiliations:** 1 Department of Mechanical Engineering, The Pennsylvania State University, University Park, PA USA; 2 Materials and Manufacturing Directorate, Air Force Research Laboratory, Wright-Patterson Air Force Base, OH USA

**Keywords:** Electrical and electronic engineering, Mechanical engineering, Polymers

## Abstract

Integrated circuits utilize networked logic gates to compute Boolean logic operations that are the foundation of modern computation and electronics. With the emergence of flexible electronic materials and devices, an opportunity exists to formulate digital logic from compliant, conductive materials. Here, we introduce a general method of leveraging cellular, mechanical metamaterials composed of conductive polymers to realize all digital logic gates and gate assemblies. We establish a method for applying conductive polymer networks to metamaterial constituents and correlate mechanical buckling modes with network connectivity. With this foundation, each of the conventional logic gates is realized in an equivalent mechanical metamaterial, leading to soft, conductive matter that thinks about applied mechanical stress. These findings may advance the growing fields of soft robotics and smart mechanical matter, and may be leveraged across length scales and physics.

## Abstract (web summary)

A method to cultivate decision-making in soft materials would provide a key step to autonomous engineered matter. Here, the authors report a class of conductive polymer-based mechanical metamaterials that process information by digital logic and permit logic gate assembly.

## Introduction

Structurally and materially compliant integrated circuits with reconfigurable electrical functions are an essential foundation for human-machine interfaces, soft robotics, and other future electronics that will serve medicine, science, engineering, and industry. These compliant conductors alleviate concerns of failure traditionally encountered when conventional metallic conductors are subjected to mechanical stress^1–5^. Liquid metal is often leveraged in compliant integrated circuits for the high conductivity and reversible self-healing behavior realized by liquid metal-based circuit interconnects^6–8^. Two-phase materials that combine conductive microparticles with a polymer substrate also support electrical function when subjected to large strains, while moreover being amenable to diverse fabrication practices^9–12^. To design the substrate for reconfiguration of a compliant integrated circuit, programmable mechanical deformation is often a candidate for reversible shape change^3,4^. For instance, kirigami^13^ and origami^14^ have inspired substrates that fold to tailor electrical behavior^15^. Elastic instabilities are also considered to transition electrical states in response to mechanical^16^ and thermal^17^ stresses.

Reconfiguration of electrical networks is indeed the basis of digital logic, which is integral for information processing in modern computers as well as in the human brain^18^. The search for logic-based information processing in artificial materials has led to concepts of discrete transmittance outputs resulting from elastic wave logic operations in mechanical metamaterials^19^ and from infrared wave logic operations in photonic metamaterials^20^. Logic functions in soft matter have also been emulated by high contrast colorimetric outputs from thermochromic elastomers subjected to input pressure states^21^.

Recent progress on embodiments of mechanologic provide another modality of information processing in soft matter. By the concept of mechanologic, a digital bit is abstracted as a reversible, mechanical, or material configuration^22,23^. Recently, discrete shape reconfigurations are cultivated by elastic beam buckling^24,25^. In formulations of buckling-based mechanologic, the two statically stable configurations represent the physical abstraction of discrete digital bits. Together, the digitized bits associated with mechanical buckling modes facilitate logic operations according to the design and assembly of the switch-able constituents^26^. This stratagem has been shown for mechanically^27^, chemically^28^, and humidity^29^ triggered elastic instabilities. Controller signaling for pressurized soft robots has also been demonstrated through mechanologic-based signal processing, suggesting one means for soft matter autonomy^30^. Yet, current embodiments of logic processing of mechanical stress inputs lack digital electrical outputs, which limits means to communicate with actuation and sensory mechanisms that may require electrical feedback to function^26,27^. Similar to anatomical realizations of intelligence and information processing to monitor and transmit sensory and motor impulses^31^, a means to cultivate decision*-*making capability in mechanically-robust compliant materials would provide a significant step towards autonomous soft matter able to assess and react to mechanical-electrical stimuli in a dynamic environment.

This report introduces a class of soft, conductive mechanical metamaterials with programmable elastic instabilities that function as electronic logic gates able to perform digital computations on mechanical stress input combinations. Here, we exploit reversible compact-to-deployed state transitions in conductive mechanical metamaterials through a strategic patterning of compliant substrate and soft, conductive material networks. We demonstrate physically reconfigurable metamaterials that respond to mechanical stress in discrete modes leading to changes in the compliant conductor network to realize all digital logic functions and logic assemblies.

## Results

### A conductive mechanical metamaterial digital switch

**Fig. 1:**
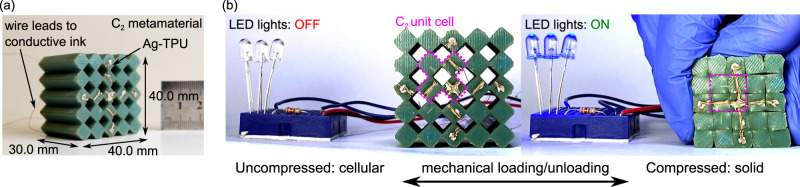
Compaction principle of electrical network switching in soft, conductive mechanical metamaterials. **a** Introduction of a metamaterial composed of C_2_ unit cell with conductive Ag-TPU trace. **b** C_2_ metamaterial in series with a power source and LED array to illustrate switching functionality. Uncompressed: Open circuit, LED off. Compacted: Closed circuit, LED on.

The metamaterials employ a constant cross-section consisting of a fundamental square tiling tessellation with square voids at the intersections. Shim et al.^32^ report that this geometry exhibits a negative Poisson’s ratio and a fully compacted state wherein the porosity of the compacted material is 0% in the ideal case of kinematic reconfiguration. In Fig. 1, we integrate this substrate architecture with conductive ink patterns that leverage the deformation of the metamaterial to reconfigure electrical circuits in discrete modes. Through this report, we build upon such fundamental building block to formulate all digital logic gates and to establish gate assembly and interconnection methods.

The elastomeric material substrates considered here are fabricated by casting liquid urethane rubber (Smooth-On VytaFlex 60) in 3D-printed molds (FlashForge Creator Pro) containing the negative of the sample architecture. The substrates also include surface channels in which the conductive networks are applied, Fig. 1(a). The conductive ink utilized in the channels is a composite containing silver (Ag) microflakes (Inframat Advanced Materials, 47MR-10F) and thermoplastic polyurethane (TPU) elastomer (BASF Elastollan Soft 35 A) to create conductive percolating networks. Copper wire leads that pass through the specimen via internal channels allow for electrical connections from an external voltage source or readout to the Ag-TPU trace terminals. Complete sample fabrication and characterization details are given in the Supplementary Information.

The metamaterials studied in this report are referred to by the unit cell that constructs the constant cross-section architecture. In Fig. 1(a), the metamaterial is assembled from C_2_ unit cells to be characterized more fully in the subsequent section of this report. An Ag-TPU trace pattern is applied to the elastomeric substrate. An electrical power supply and LED array are interfaced in series with the silver trace terminals at the sample leftmost and rightmost sides, respectively. By applying uniaxial displacement to the top of the metamaterial, the sample deforms into a fully compacted solid square Fig. 1(b). As a result, the Ag-TPU network closes through self-contact, passing electrical current through the LED array. Once the mechanical load is released, the Ag-TPU network opens and the LED array turns off again. Continued cycling of the metamaterial repeats the electrical switch behavior. The principle of reversible mechanical-electrical switching demonstrated in Fig. 1 is harnessed in this report to formulate all logic gate operations and logic assembly methods in a class of soft, conductive mechanical metamaterials.

**Fig. 2:**
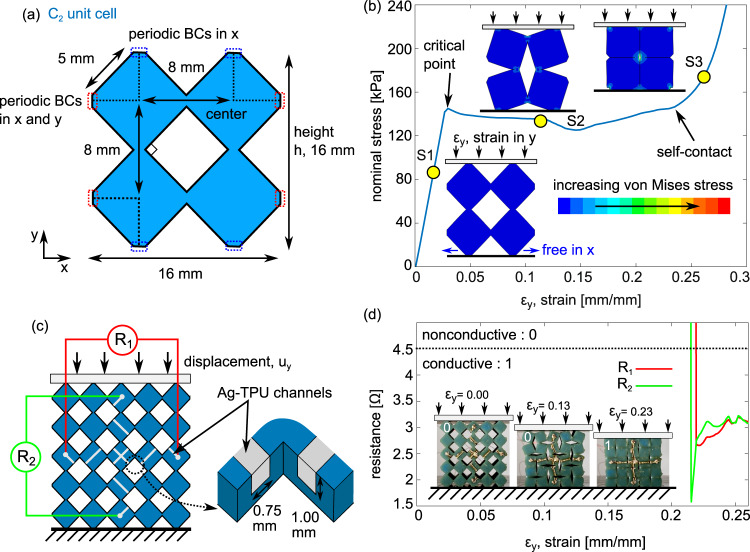
Bit abstraction via open/closed circuit. **a** C_2_ unit cell dimensions with periodic boundary conditions. **b** Simulated mechanical response of C_2_ unit cell featuring three regimes of general behavior and shape change. **c** Schematic of C_2_ metamaterial with Ag-TPU biaxial switch network. Resistance measurements R_1  _and R_2_ are monitored across two terminal pairs. **d** Experimental resistance measurement with photos depicting the deformations in the three compression regimes.

To characterize the elastic buckling deformation of the metamaterial unit cells, we develop a finite element model that simulates the material response observed in experiments (see Supplementary Information). The C_2_ unit cell geometry is shown in Fig. 2(a). The unit cell consists of two rows and two columns of a continuous square tiling pattern. A hyperelastic Neo–Hookean material model is employed on the 2D cross-section, considering plane strain assumptions and accounting for nonlinear deformations under quasi-static, uniaxial compressive displacement in the vertical direction $u_y$. Periodic boundary condition displacements in $x$ and $y$ are applied on the nodes highlighted with the dashed blue and red lines in Fig. 2(a).

The mechanical response for the C_2_ unit cell illustrates three regimes during uniaxial compression, Fig. 2(b). Linear elastic response (label S1) occurs for small applied strains $\varepsilon _y = u_y/h$, such as <2% strain. At a critical strain, namely $\varepsilon _{critical}$ = 0.0279 in Fig. 2(b), a second regime (label S2) displays large decrease in uniaxial stiffness and a rotational behavior of the unit cell. With further increase in strain, a final transition occurs wherein self-contact and near-total compaction of the unit cell is achieved (label S3), leading to increase in uniaxial stiffness. For unit cell C_2_, the self-contact strain is $\varepsilon _{contact}$ = 0.23. The compact state in Fig. 2(b) represents the compacted shape of the experimental sample in Fig. 1(c) that closes the electrical circuit by self-contact of the Ag-TPU trace.

To demonstrate the relationship between the mechanical and electrical behavior by the compaction principle, we design a biaxially functional electrical switch Ag-TPU trace pattern on the surface of a metamaterial composed of C_2_ unit cells, Fig. 2(c). The metamaterial is loaded quasi-statically in the vertical direction between rigid aluminum platens using a load frame (see Supplementary Information). Voltage divider circuits measure the electrical resistances R_1_ and R_2_ across the horizontal and vertical Ag-TPU terminal pairs, respectively.

Using the C_2_ unit cell geometry and the Ag-TPU network on the sample, the metamaterial functions as a strain gated switch. For instance, Fig. 2(d) shows that the resistances R_1_ and R_2_ through the Ag-TPU trace pairs exhibit a nonconductive, infinite resistance that represents a 0 digital readout. Once the contact strain $\varepsilon _{contact}$ = 0.23 is reached, the networks close as observed by the sudden drops in R_1_ and R_2_ to mean resistances near 3 Ω. The closed electrical connections therefore represent a 1 digital readout. A supporting kinematic model of the C_2_ unit cell confirms self-contact is induced near applied strain of $\varepsilon _{contact}$ = 0.201, Supplementary Fig. 2(a) (see Supplementary Information). The similarity between the sudden changes in resistances R_1_ and R_2_ illustrates that the electrical connections may be achieved biaxially despite uniaxial mechanical input. These results introduce a technique to exploit transitions between uncompressed and compacted states of soft, conductive mechanical metamaterials to govern digital electrical signals.

### Method for digital logic in conductive mechanical metamaterials

**Fig. 3:**
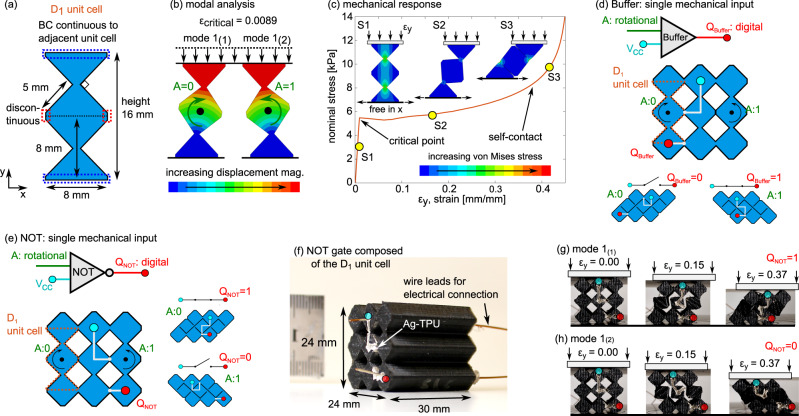
Bit abstraction via buckling direction. **a** D_1_ unit cell dimensions with boundary conditions. **b** Modal analysis of D_1_ unit cell with two deformation states, mode 1_(1)_ and mode 1_(2)_. **c** Simulated mechanical response of D_1_ unit cell featuring three deformation regimes. **d** Schematic of Buffer gate as direct electrical switch governed by a single mechanical rotation input, corresponding to the buckling mode. **e** Schematic and (**f**) experimental image of the NOT gate with one mechanical input rotation (green) and one digital output terminal (red) Q_NOT_ on the metamaterial composed of D_1_ unit cells. The powered input terminal is the cyan node, $V_{cc}$. Photos of the NOT gate under uniaxial compression as it exhibits buckling (**g**) mode 1_(1)_ and (**h**) mode 1_(2)_, along with the corresponding digital outputs Q_NOT_ (red node).

We leverage this foundation to create logic gates controlled by mechanical deformation. Metamaterials composed from C_2_ unit cells may exhibit two rotations in the lowest order buckling mode, Supplementary Fig. 3. This limited switch behavior is a result of the distinct buckling modes and the choice of Ag-TPU network. As a result, we introduce a second unit cell termed D_1_ that exploits a discontinuity with laterally adjacent unit cells in the left/right directions, Fig. 3(a).

A modal analysis of the D_1_ unit cell is carried out to characterize the lowest order elastic buckling behavior. The unit cell top and bottom boundaries are relatively displaced with one side free to laterally displace in a given simulation. As shown in Fig. 3(b), the D_1_ unit cell exhibits a buckling mode at a critical strain of 0.0089. Two states of deformation may occur for the same critical strain. Mode 1_(1)_ exhibits a clockwise rotation of the central bulk material block, while mode 1_(2)_ exhibits a counterclockwise rotation. Realization of these modes is dependent upon the vector of shear and uniaxial compression applied respecting the horizontal mirror plane. Uniaxial compression simulations are conducted to determine the mechanical properties of the D_1_ unit cell. The results in Fig. 3(c) likewise reveal 3 collapse regimes similar in qualitative trend and interpretation with behavior of the C_2_ unit cell in Fig. 2(b). The D_1_ unit cell exhibits a lower critical strain $\varepsilon _{critical}$ = 0.0089 and a greater self-contact strain $\varepsilon _{contact}$ = 0.40 compared with the C_2_ unit cell. A noticeable difference is also evident in the compacted states. Namely, the D_1_ unit cell compacts into a parallelogram cross-section Fig. 3(c), compared with the square compacted state for the C_2_ unit cell Fig. 2(b). Consequently, the metamaterial unit cells permit unique approaches to Ag-TPU trace networking to realize reconfigurable electrical interconnects.

Digital logic gates are provided with digital inputs and pass digital outputs. Our formulation of digital logic intrinsically couples discrete mechanical behavior with electrical signals passed through the metamaterials. Here, we consider a circuit powering node provided to an input terminal of the unit cell. The input node is highlighted in cyan color and labeled as $V_{cc}$ on the NOT logic gate material shown in Fig. 3(d). The output node is highlighted in red color. To provide the digital logic operations, we exploit the mechanical buckling modes, considering counterclockwise rotation of the D_1_ unit cell as a digital input of 1 and clockwise rotation as a digital input of 0, according to electrical conduction through the D_1_ unit cell layer. The fundamental Buffer gate exemplifies this digitizing behavior as seen in Fig. 3(d), where counterclockwise rotational collapse of the D_1_ unit cell metamaterial outputs Q_Buffer_ of 1, and Q_Buffer_ of 0 for clockwise rotation. The Buffer metamaterial gate is realized by three D_1_ unit cells that are tiled together, keeping the discontinuity at the central bulk material block. The Ag-TPU trace applied to the surface realizes a switch that operates in agreement to Buffer logic in accordance with buckling modes of this metamaterial.

The NOT digital logic gate is an inverter of a digital signal, converting a 0 to a 1, and vice versa. The NOT gate is thus an inverted Buffer gate. In this embodiment of soft digital logic, output signal inversion is achieved by a horizontal or vertical mirroring of the conductive network. The metamaterial realization of the NOT logic gate that we explore here is shown in Fig. 3(e) along with the corresponding logic outputs resulting from each of the clockwise and counterclockwise collapse rotations. The output digital signal Q_NOT_ is 1 for the conductive Ag-TPU network in Fig. 3(g) considering the clockwise rotation of the central bulk material blocks in the unit cells. Conversely, an output digital signal Q_NOT_ of 0 is read out for the buckling mode with counterclockwise bulk material block rotation, Fig. 3(h). As expected, these results are the opposite of the Buffer gate fundamental switch. Consequently, the digital logic realized by the discrete, modal mechanical deformation of the metamaterial in Fig. 3(f) is analogous to a digital NOT gate. Moreover, Fig. 3(g), (h) show that the collapse behavior and thus digital signaling occurs for the same values of applied strain. This indicates that exploitation of small perturbations around the bifurcation^33^, or critical strain, could be used for high sensitivity control^34^ of the combined mechanical and electrical functions of the soft, conductive logic gate.

### Formulation of all digital logic gates

**Fig. 4:**
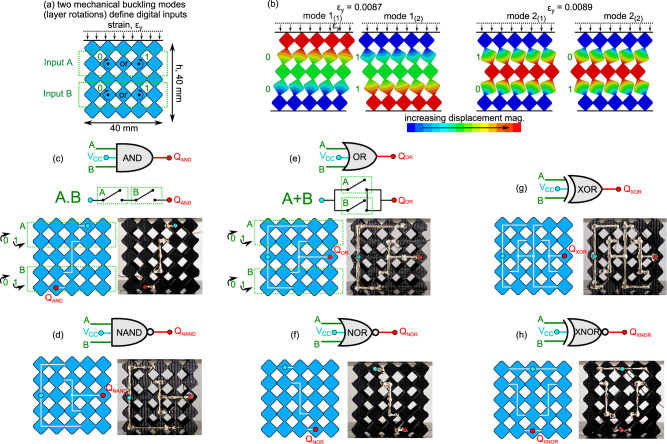
Formulation of digital logic gates in soft, conductive mechanical metamaterials. **a** Schematic of the D_1_ based metamaterials. Inputs A and B are the rotations in the first and second layers, respectively. **b** Modal analysis illustrating four possible deformation states. Schematics and experimental images of the Ag-TPU network on the metamaterials for (**c**) AND, (**d**) NAND, (**e**) OR, (**f**) NOR, (**g**) XOR, and (**h**) XNOR logic gates. AND and OR include corresponding switched circuit schematics. Each gate contains two mechanical inputs A and B (green) and one digital output Q (red). The powered input terminal is the cyan node, $V_{cc}$.

We build on this manifestation of soft, conductive matter-based digital logic to realize the remaining 6 logic gates: AND, NAND, OR, NOR, XOR, and XNOR. We use two rows of D_1_ unit cells each containing 5 unit cells to realize the metamaterial geometries shown in Fig. 4(a). The unit cells share the central row of bulk material blocks, which does not influence the layer-by-layer mechanics. We undertake a modal analysis of the materials to identify low order buckling modes. Two modes are found for critical strains 0.0087 and 0.0089, while each mode may be manifest in two deformation states, Fig. 4(b). These modes are realized by the specific vector combination of shear and uniaxial stresses applied to the top metamaterial surface. For instance, mode 1_(1)_ requires shear to the right and then uniaxial compression, while mode 2_(1)_ requires uniaxial compression then shear to the left near the critical strain, and vice versa for the mode 1_(2)_ and mode 2_(2)_. Thus, the logic inputs are represented by the layer-by-layer rotations, illustrated in Fig. 4(a) with green labels. Input A of the soft, conductive logic gate indicates the rotation of the top layer of unit cells, while Input B indicates the rotation of the bottom layer of unit cells. This introduces a unique technique to control mechanical digital inputs through stress vectors and discrete self-contact states in soft mechanical metamaterials^35^.

The Ag-TPU trace networks required to realize the AND, NAND, OR, NOR, XOR, and XNOR gates in this formulation of soft digital logic are shown in Fig. 4(c)–(h). By using the fundamental Buffer or NOT logic gate Fig. 3(d) for each mechanical input layer and by electrically networking the outputs of each layer in series or parallel, the remaining AND, NAND, OR, NOR, XOR, and XNOR logic gates may be realized in a systematic way. For the AND and OR gates, the corresponding serial and parallel switched circuit schematics are shown in Fig. 4(c), (e). The relations between the serial (AND) and parallel (OR) switch assembly schematics and conductive trace networks are apparent considering Fig. 4(c), (e). Based on the induced mode in Fig. 4(b), the switches open or close according to (counter)clockwise assignment of (1)0 digital bits via the Ag-TPU trace networks to result in the appropriate digital output, Q. For instance, the AND gate registers Q_AND_ = 1 when both layers rotate counterclockwise, or 1. Thus, a clear analogy exists between the circuitry schematic, the Ag-TPU network topology, and the mechanical buckling deformations. The Ag-TPU trace networks shown in Fig. 4 are not unique and can be tailored to permit changing positions of the powered node (highlighted in cyan color) and output node (red color). The reading from the output nodes (red color) represent the digital output from the soft, conductive logic gate.

Gates such as the NAND in Fig. 4(d) and OR in Fig. 4(e) contain the same fundamental parallel circuit design, yet are vertically mirrored according to the horizontal midplane. The NAND network topology is obtained by applying De Morgan’s theorem, which states that the NAND gate is equivalent to an inverted OR gate. As described above related the NOT to the Buffer, the inversion operation in this embodiment of soft digital logic gates is a vertical or horizontal mirroring of the conductive trace network. As a result, De Morgan’s theorem is readily applied to create the NAND metamaterial gate Fig. 4(d) via the base construct of the OR metamaterial gate Fig. 4(e). DeMorgan’s theorem is also applied to design the NOR gate Fig. 4(f) from the embodiment of the AND gate Fig. 4(c). Finally, the XOR metamaterial gate Fig. 4(g) and XNOR gate Fig. 4(h) are obtained through a serial combination of OR-NAND gates and parallel combination of AND-NOR gates, respectively. Such design protocol is directly analogous to the formulation of conventional switched electrical networks that provide digital logic functionality.

**Fig. 5:**
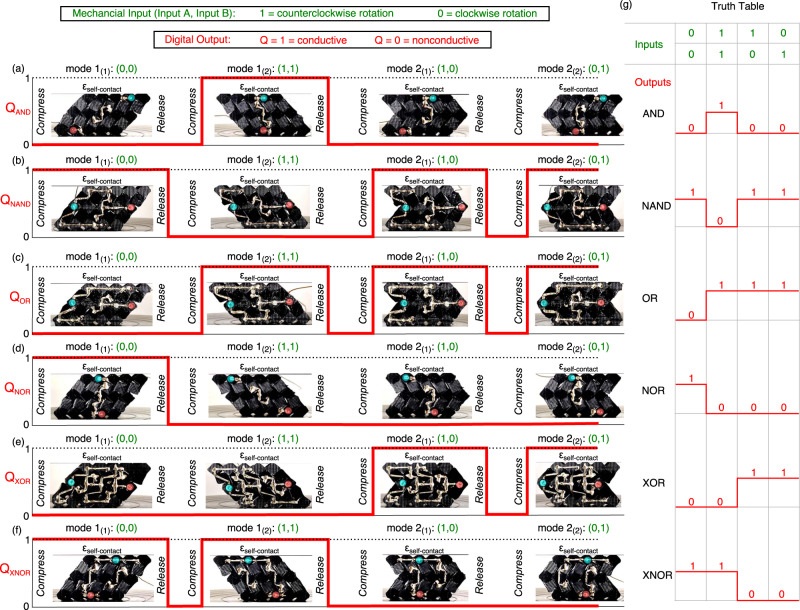
Processing all digital logic operations in soft, conductive mechanical metamaterials. Logic gate digital output Boolean response at each of the four compact states for the (**a**) AND, (**b**) NAND, (**c**) OR, (**d**) NOR, (**e**) XOR, and (**f**) XNOR logic gate. The respective mechanical inputs (green) for each of the deformation states directly relate to (**g**) the logic gates truth table inputs.

To validate the functioning of the logic gates, samples are fabricated and compressed using the experimental setup in Supplementary Fig. 1(b). Each of the buckling modes is achieved through cyclic loading and unloading. Figure 5 compiles photographs of deformed modes, resulting Ag-TPU network connectivity, and associated truth tables.

The experimental digital outputs for each buckling mode behavior agree with the respective truth table entry for the ideal logic gates, Fig. 5. For instance, the AND gate in Fig. 5(a) provides a digital output Q_AND_ = 1 for mode 1_(2)_ when both mechanical inputs are counterclockwise rotations, and a digital output of Q_AND_ = 0 for all other combinations. The Supplementary Video 1 shows the transitions among the digitized, modal inputs, and the digital outputs, with an LED array indicator for visualization.

## Discussion

This foundation is extensible to the formulation of logic gate combinations used in integrated circuits. For instance, Supplementary Fig. 9 demonstrates a three-input logic gate combination of OR-NAND on a three layer metamaterial. To create the OR-NAND network topology, a negated OR gate Fig. 4(e) interfaces with the NAND gate parallel switch Fig. 4(d). Interestingly, by the unique D_1_ unit cell design explored here, metamaterial gates with *n* layers possess 2^*n*^ self-contacting buckling modes, in agreement with the number of binary input combinations for the *n* input gate assembly. This differentiating characteristic allows for a systematic metamaterial design process to generate arbitrary gate combinations via strategic conductive trace network topologies (see Supplementary Information), although the platforms are not as much assembled from modules as they are designed into a monolithic metamaterial circuit. Explorations of modularly assembled soft logic gates using our mechanical metamaterial modules may take inspiration from mechanical signal transmission concepts^26,27^ as well as techniques explored with pneumatic-mechanical realizations of soft logic^30^. Although the logic gates formulated here do not compete with the speed and component scaling of conventional digital microprocessors, the conductive metamaterial-based information processing may provide essential robustness of function and material compatibility for future autonomous soft matter that emulate numerous biological systems.

By establishing interfaces between soft mechanical metamaterials and digital logic gating, this work formulates a general method to synthesize discrete mechanical configurations with conductive networks to govern digital electrical outputs. As one manifestation of soft, smart matter^36^, the mechanical metamaterial building blocks exploited in this work may be considered as building blocks for future soft matter assemblies, such as to realize autonomous soft machines with integrated sensory, actuation, and decision-making functionalities^37^. The concepts embodied in this report are also free of length-scale dependence, by leveraging nominally kinematic rearrangement of cellular solids^32^, and suggest one means to process mechanical stress inputs without need for periodic sensor observation. Consequently, physical embodiments of these principles may be exploited for low-power information processing in fields as diverse as DNA origami^38^, electro-optical filters^39^, reconfigurable antenna^40^, and more.

## Methods

### Specimen fabrication methods

#### Metamaterial substrate fabrication

The elastomeric metamaterial substrates are fabricated by casting liquid urethane rubber (Smooth-On VytaFlex 60) in a two-part mold. The mold parts are designed in CAD software SOLIDWORKS and 3D-printed (FlashForge Creator Pro) with acrylonitrile butadiene styrene (ABS) filament. When assembled together, the two-part mold realizes the negative of the metamaterial substrate shape. The liquid urethane rubber utilized in this research is a two-part material, A and B parts that are initially mixed in a 1 A:1 B volume ratio and stirred by hand for 2 min. After the material is poured into the mold, it is cured for 24 h. The sample is then carefully demolded and prepared for testing. Metamaterials using C_2_ and D_1_ unit cells are prepared utilizing this procedure.

#### Conductive ink fabrication and deposition

The conductive ink utilized in the channels is a composite containing 35% (volume %, v%) silver (Ag) microflakes (Inframat Advanced Materials, 47MR-10F) and 65% (v%) thermoplastic polyurethane (TPU) elastomer (BASF Elastollan Soft 35 A). To begin processing the conductive ink, Ag microflakes are first mixed in a glass vial with sufficient N-Methyl-2-pyrrolidone (NMP) solvent, and sonicated (Branson M2800 Ultrasonic Cleaner) for 60 min. TPU is then added to the Ag-NMP mixture, and planetary mixed (KK 300SS Mazerustar) at 225 $\times$g for 2-minute increments. The planetary mixing process is repeated three times with gentle hand stirring in between to ensure walls of the vial do not collect excess Ag or TPU particles. After the mixture is given 48 h for the NMP to evaporate at room temperature, the Ag-TPU ink is ready for application.

Enamel-coated copper wire (22 gauge) leads are passed through small molded-in 1.50 mm diameter channels in the substrate to terminate at the front cross-sections of the metamaterials. Metamaterial photographs in Figs. 1 and 3 of the main text and in the Supplementary Video 1 show more clearly how the copper wires terminate at the front cross-sections. Enamel is only removed from both extremities of the copper wire ends to eliminate trace electrical shorting along the wire length. To secure the wire leads in the channels through the substrates, a small amount of silicone adhesive (DAP All-Purpose) is deposited by syringe in the internal channels through the rear-facing cross-section. Using a 3.0 cc dispensing syringe, the surface channels on the substrate are filled with Ag-TPU ink and allowed to cure around the copper wire leads for 24 h.

### Experimental characterization methods

The unit cells C_2_ and D_1_ and metamaterials assembled from the unit cells are examined using distinct experimental methods by virtue of the unique mechanical behaviors that motivate distinct electrical circuit switching methods. The specimens are quasi-statically and vertically displaced $u_y$ by a polished, rigid platen from a load frame (ADMET eXpert 5600) at a loading rate of 0.5 mm min^−1^, shown in Supplementary Fig. 1. A laser displacement sensor (Micro-Epsilon optoNCDT ILD1700-200) is attached to the load frame to measure vertical displacements $u_y$ of the platen. For metamaterial C_2_, the bottom specimen surface rests on a polished, fixed aluminum plate, Supplementary Fig. 1(a). As a result, for C_2_ the top and bottom surfaces of the specimen have limited motion in lateral displacements $u_x$ due to friction against the platen and plate. The uniaxial applied strain is calculated from $\varepsilon _y = u_y/h$ where $h$ is the macroscopic height of the metamaterial sample or unit cell.

For metamaterial D_1_, the bottom surface is presumed to slide in the simulations reported in the main text. To permit lateral sliding of metamaterials in the experimental characterization, we place a polished, rigid aluminum plate on smooth cylindrical shafts that act as a rolling surface for the bottom of the D_1_ samples, Supplementary Fig. 1(b). Such a setup provides uninhibited lateral displacements $u_x$. The direction of cross-section rotation in experiments may be governed at critical points by manual methods in order to achieve all buckling modes, since only the lowest order mode occurs naturally in the absence of additional control measures. Voltage divider circuits are used to measure resistance through the Ag-TPU ink traces with a 1.30 Ohm reference resistor, where the electrical resistances R_1_ and R_2_ are evaluated across the horizontal and vertical Ag-TPU terminal pairs, respectively. Data is collected through an acquisition system (NI USB 6341 Multifunction DAQ) and analyzed in MATLAB.

### Finite element modeling methods

ABAQUS Linear Buckling Perturbation and Dynamic-Implicit simulations are conducted with 2D plane strain models based on the constant material cross section. For the Linear Buckling Perturbation simulations, a subspace eigensolver is used to determine the low-order buckling modes. For the nonlinear Dynamic-Implicit step, a time period that is proportional to the 0.5 mm.min^−1^ quasi-static experimental load frame loading rate is used. A hyperelastic Neo–Hookean material model is employed with Young’s modulus of 2.07 MPa and Poisson’s ratio of 0.499 for the samples considered here. Free quadrilateral elements are used for all geometry meshes with seed sizes proportional to the smallest geometric feature with at least 0.25 ratio of element characteristic dimension to substrate feature size.

For the C_2_ unit cell, periodic boundary conditions are imposed on the outermost nodes while the bottom boundaries are fixed in $y$ and free to slide in $x$. Shown in Fig. 2(a) in the main text, the left-most and right-most boundary nodes (outlined in red) are constrained to mirror the displacements of nodes in $x$ and $y$, and similarly the top-most and bottom-most boundary nodes (outlined in blue) are constrained to mirror displacements only in $x$. For the D_1_ unit cell and metamaterial assemblies the boundaries are fixed in $y$ and free to slide in $x$ at the bottom edge.

By applying compressive displacements $u_y$ at the top boundary of the unit cells and metamaterial assemblies, we acquire the buckling modes as well as the mechanical properties through the Linear Perturbation and Dynamic-Implicit simulations, respectively.

### Statistics and reproducibility

All experiments and simulations are conducted a minimum of 6 times to confirm repeatability of trends and quantities.

## Supplementary information

Supplementary Information

Supplementary Movie 1

## Data Availability

Models, codes, and datasets generated during and/or analyzed during the current study are available from the corresponding author on request. All data is available in the main text or the Supplementary Information.
