# Supplementary material for: Digital logic gates in soft, conductive mechanical metamaterials
Source: Nat Commun. 2021 Mar 12;12:1633. doi: 10.1038/s41467-021-21920-y (PMC7954845; doi:10.1038/s41467-021-21920-y)
Supplement: Supplementary file 1 — Supplementary Information [file 41467_2021_21920_MOESM1_ESM.pdf]

Supplementary Information for
Digital Logic Gates in Soft, Conductive Mechanical Metamaterials

Charles El Helou, Philip R. Buskohl, Christopher E. Tabor, and Ryan L. Harne

Correspondence to: ryanharne@psu.edu

1 Methods

1. Specimen fabrication methods

1.1 Metamaterial substrate fabrication

The elastomeric metamaterial substrates are fabricated by casting liquid urethane rubber (Smooth-On VytaFlex 60) in a two-part mold. The mold parts are designed in CAD software SOLIDWORKS and 3D-printed (FlashForge Creator Pro) with acrylonitrile butadiene styrene (ABS) filament. When assembled together, the two-part mold realizes the negative of the metamaterial substrate shape. The liquid urethane rubber utilized in this research is a two-part material, A and B parts that are initially mixed in a 1A:1B volume ratio and stirred by hand for 2 minutes. After the material is poured into the mold, it is cured for 24 hours. The sample is then carefully demolded and prepared for testing. Metamaterials using C2 and D1 unit cells are prepared utilizing this procedure.

1.2 Conductive ink fabrication and deposition

The conductive ink utilized in the channels is a composite containing 35% (volume %, v%) silver (Ag) microflakes (Inframat Advanced Materials, 47MR-10F) and 65% (v%) thermoplastic polyurethane (TPU) elastomer (BASF Elastollan Soft 35A). To begin processing the conductive ink, Ag microflakes are first mixed in a glass vial with sufficient N-Methyl-2-pyrrolidone (NMP) solvent, and sonicated (Branson M2800 Ultrasonic Cleaner) for 60 minutes. TPU is then added to

the Ag-NMP mixture, and planetary mixed (KK 300SS Mazerustar) at 225 $\times$g for 2-minute increments. The planetary mixing process is repeated 3 times with gentle hand stirring in between to ensure walls of the vial do not collect excess Ag or TPU particles. After the mixture is given 48 hours for the NMP to evaporate at room temperature, the Ag-TPU ink is ready for application.

Enamel-coated copper wire (22 gauge) leads are passed through small molded-in 1.50 mm diameter channels in the substrate to terminate at the front cross-sections of the metamaterials. Metamaterial photographs in Figures 1 and 3 of the main text and in the Supplementary Video 1 show more clearly how the copper wires terminate at the front cross-sections. Enamel is only removed from both extremities of the copper wire ends to eliminate trace electrical shorting along the wire length. To secure the wire leads in the channels through the substrates, a small amount of silicone adhesive (DAP All-Purpose) is deposited by syringe in the internal channels through the rear-facing cross-section. Using a 3.0 cc dispensing syringe, the surface channels on the substrate are filled with Ag-TPU ink and allowed to cure around the copper wire leads for 24 hours.

2. Experimental characterization methods

The unit cells C_2 and D_1 and metamaterials assembled from the unit cells are examined using distinct experimental methods by virtue of the unique mechanical behaviors that motivate distinct electrical circuit switching methods. The specimens are quasi-statically and vertically displaced u_y by a polished, rigid platen from a load frame (ADMET eXpert 5600) at a loading rate of 0.5 mm min^{-1}, shown in Supplementary Figure 1. A laser displacement sensor (Micro-Epsilon optoNCDT ILD1700-200) is attached to the load frame to measure vertical displacements u_y of the platen. For metamaterial C_2, the bottom specimen surface rests on a polished, fixed aluminum

plate, Supplementary Figure 1(a). As a result, for C_2 the top and bottom surfaces of the specimen have limited motion in lateral displacements u_x due to friction against the platen and plate. The uniaxial applied strain is calculated from $\varepsilon_y = u_y / h$ where h is the macroscopic height of the metamaterial sample or unit cell.

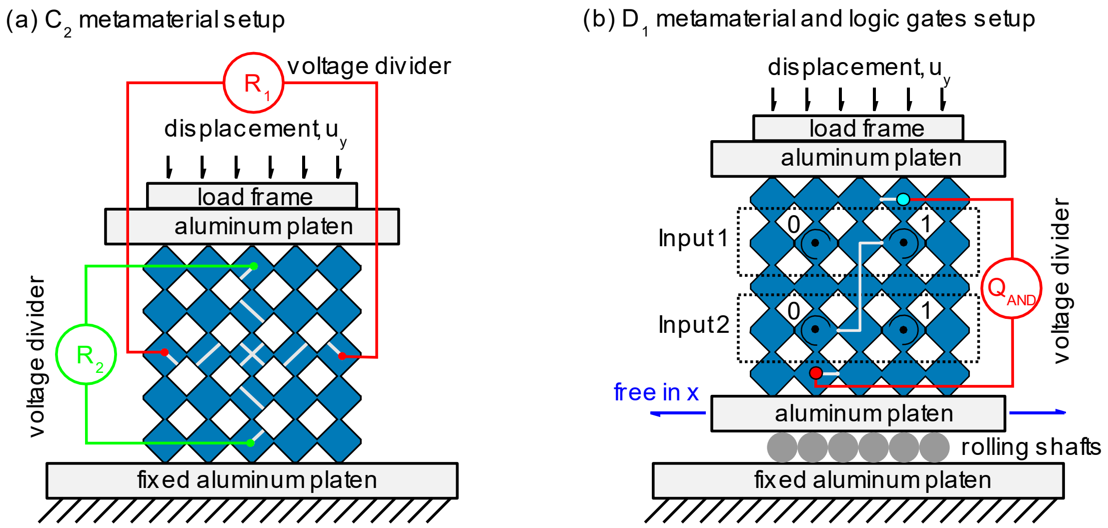

Supplementary Figure 1. Schematic of the experimental loading setup utilized for metamaterials composed of (a) C_2 and (b) D_1 unit cells. The AND gate circuitry is depicted in the schematic, yet such setup is used for all logic gates.

For metamaterial D_1, the bottom surface is presumed to slide in the simulations reported in the main text. To permit lateral sliding of metamaterials in the experimental characterization, we place a polished, rigid aluminum plate on smooth cylindrical shafts that act as a rolling surface for the bottom of the D_1 samples, Supplementary Figure 1(b). Such a setup provides uninhibited lateral displacements u_x. The direction of cross-section rotation in experiments may be governed at critical points by manual methods in order to achieve all buckling modes, since only the lowest

order mode occurs naturally in the absence of additional control measures. Voltage divider circuits are used to measure resistance through the Ag-TPU ink traces with a 1.30 Ohm reference resistor, where the electrical resistances R_1 and R_2 are evaluated across the horizontal and vertical Ag-TPU terminal pairs, respectively. Data is collected through an acquisition system (NI USB 6341 Multifunction DAQ) and analyzed in MATLAB.

3. Finite element modeling methods

ABAQUS Linear Buckling Perturbation and Dynamic-Implicit simulations are conducted with 2D plane strain models based on the constant material cross section. For the Linear Buckling Perturbation simulations, a subspace eigensolver is used to determine the low-order buckling modes. For the nonlinear Dynamic-Implicit step, a time period that is proportional to the 0.5 $mm.min^{-1}$ quasi-static experimental load frame loading rate is used. A hyperelastic Neo–Hookean material model is employed with Young's modulus of 2.07 MPa and Poisson's ratio of 0.499 for the samples considered here. Free quadrilateral elements are used for all geometry meshes with seed sizes proportional to the smallest geometric feature with at least 0.25 ratio of element characteristic dimension to substrate feature size.

For the C_2 unit cell, periodic boundary conditions are imposed on the outermost nodes while the bottom boundaries are fixed in y and free to slide in x. Shown in Figure 2(a) in the main text, the left-most and right-most boundary nodes (outlined in red) are constrained to mirror the displacements of nodes in x and y, and similarly the top-most and bottom-most boundary nodes (outlined in blue) are constrained to mirror displacements only in x. For the D_1 unit cell and metamaterial assemblies the boundaries are fixed in y and free to slide in x at the bottom edge.

By applying compressive displacements u_y at the top boundary of the unit cells and metamaterial assemblies, we acquire the buckling modes as well as the mechanical properties through the Linear Perturbation and Dynamic-Implicit simulations, respectively.

4. C_2 unit cell analysis

This section includes supporting analysis conducted on the C_2 metamaterial unit cell including kinematic modeling and modal analysis.

4.1 Kinematic model

To understand the deformation of structures comprised from the continuous C_2 unit cell, we develop a kinematic model to characterize the rotation of unit cell constituents observed experimentally and computationally. The model is particularly utilized to predict the strain ε_y at self-contact $\varepsilon_{contact}$, at which electrical connection is presumed to occur. The geometry seen in Supplementary Figure 2(a) is used to model the system. The C_2 unit cell is comprised of four tessellated material blocks labeled at the respective block centers by coordinates O_n for the nth block. Each block includes four coordinates at the center of the material sides labeled as $A_{n,m}$ for the mth side of the nth block, and four coordinates at the center of the square voids adjacent to each side labeled as $B_{n,m}$.

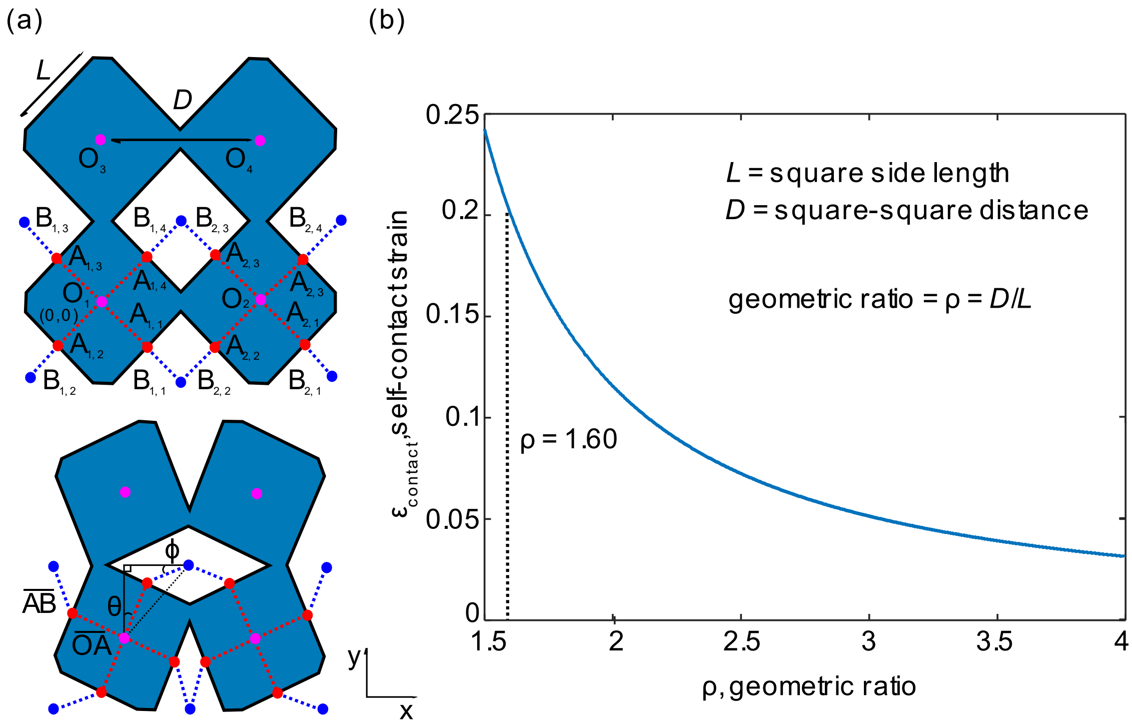

Supplementary Figure 2. (a) Schematics employed to derive the kinematic model for the C_2 unit cell rotation. (b) Relationship between the geometric ratio and the self-contact strain.

We assume that the material preserves shape of the solid bulk material blocks, so that the lengths $\left|\overline{OA}\right|$ and $\left|\overline{AB}\right|$ remain constant throughout the full rotation from uncompressed to compacted. The Equations (1) and (2) define $\left|\overline{OA}\right|$ and $\left|\overline{AB}\right|$, respectively. The parameters D and L are identified as unit cell dimensions in Supplementary Figure 2(a).

$$\left|\overline{OA}\right| = \frac{\sqrt{2}D - L}{2} \tag{1}$$

$$\left|\overline{AB}\right| = \frac{L}{2} \tag{2}$$

Since the C_2 unit cell has a single degree of freedom, we use the rotation angle θ as the state variable. The angle ϕ is then defined in terms of the state variable, as shown in Equation (3), assuming that the voids are regular squares.

$$\phi = \frac{\pi}{4} - \sin^{-1}\left(\frac{2\left|\overline{OA}\right|}{L}\sin\left(\frac{\pi}{4} - \theta\right)\right) \tag{3}$$

We utilize Equations (1), (2), and (3) to determine the vectors $\overline{OA}$ and $\overline{AB}$ for a specific value of θ induced by applied disturbances, such as the applied displacements in the computational and experimental characterization, Sec. 1. When adjacent solid material blocks of the C_2 unit cell contact at critical values of rotational angle θ, we refer to this event as "self-contact". At self-contact, we find that $A_{1,1} = A_{2,2}$ and $\phi = 0$. These relations are used to solve for the self-contact rotation $\theta_{contact}$. Likewise, the calculations lead to knowledge of the net height of the specimen and thus the self-contact strain $\varepsilon_{contact}$. In Supplementary Figure 2(b) the relationship between the self-contact strain $\varepsilon_{contact}$ and the geometric ratio ρ of the C_2 unit cell is presented. The strain at contact decreases as the distance between the voids increases. For instance, at $\rho = 1.6$ a self-contact strain of $\varepsilon_{contact} = 0.201$ is determined. In this report, all samples fabricated of the C_2 and D_1 unit cells employed a $\rho = 1.6$ geometric ratio.

4.2 Modal analysis

We undertake a modal analysis of the C_2 unit cell via the finite element method in ABAQUS to illuminate buckling modes that govern connectivity of the sample Ag-TPU traces when the metamaterials are subjected to uniaxial compression. Periodic boundary conditions are imposed

on the lateral boundaries to study nominal unit cell mechanical behavior. As shown in Supplementary Figure 3(a), one buckling mode at a critical uniaxial strain ε_y of 0.0279 is determined with two deformation states. The two states of deformation for the lowest order buckling mode for C_1 are termed mode $1_{(1)}$ and mode $1_{(2)}$ and have opposing rotational behavior, Supplementary Figure 3(a). An experimental metamaterial sample composed of continuous C_2 unit cells also demonstrates the two deformation states, Supplementary Figure 3(b) and (c).

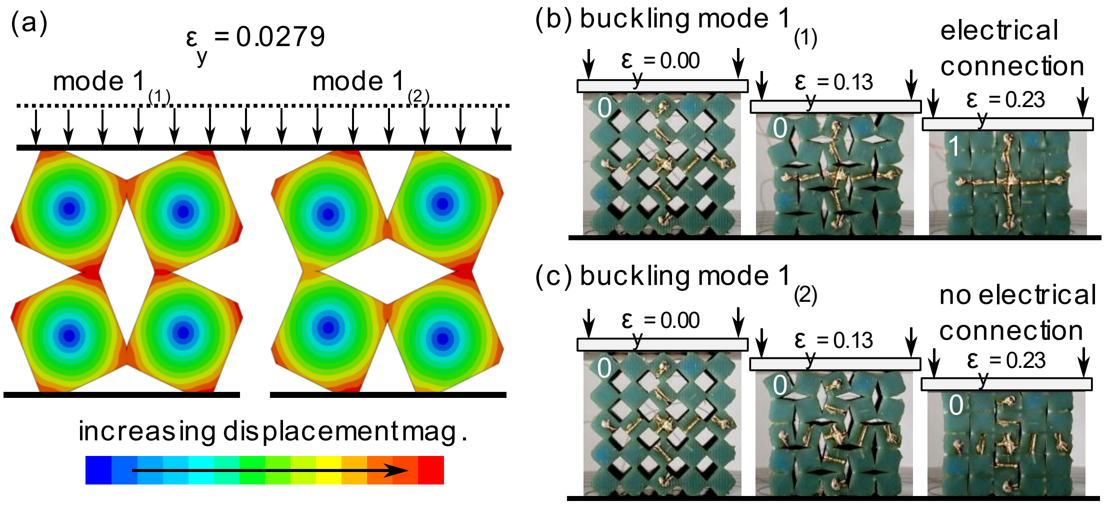

Supplementary Figure 3. (a) Modal analysis of the lowest order buckling mode of the C_2 metamaterial unit cell. Experimental images of a metamaterial assembled from C_2 unit cells exhibiting (b) mode $1_{(1)}$ and (c) mode $1_{(2)}$.

5. D_1 unit cell analysis

Here we array the D_1 unit cell in three layers to create a metamaterial assembly seen in Supplementary Figure 4(a). The modal analysis uncovers the three lowest order buckling modes with similar values of critical strain at 0.0089, 0.0091, and 0.0092 strain, Supplementary Figure 4(b). Considering the three serially assembled layers of the metamaterial in Supplementary Figure

4(a) and the fact that each row may adopt one of two central block rotations, the metamaterial may exhibit at any time 1 of (2^3) 8 deformation states. We adopt the NOT gate elemental circuit utilizing Ag-TPU ink traces to monitor the occurrence of the sample modal response. The undeformed metamaterial assembly is shown in Supplementary Figure 4(c) at left.

Each layer contains inputs and a digital output. One input is a powered input node V_{cc}, shown as the cyan color highlight in Supplementary Figure 4(c). The digital inputs that govern the logic gate operation are the result of mechanical deformation and thus the rotation of the collapsing layer. Throughout this report, the counterclockwise rotation of the metamaterial unit cell layers corresponds to a digital input of 1, while the clockwise rotation of the unit cells corresponds to digital input of 0. This input notation is selected on the basis of the activation of switching behavior in the fundamental switching component that electrically connects for counterclockwise layer rotation and disconnects for clockwise layer rotation. The same convention is utilized here on the use of the Ag-TPU traces that make up the discrete switching network of electrical connections on the unit cell and metamaterial cross-sections. Alternative conventions and notations may be employed for greater adaptability of digital switching and logic operation based on alternative unit cell mechanical behaviors. As the material is quasi-statically compressed, the digital output Q is monitored at the Ag-TPU trace point shown as the red, green and blue color highlight in Supplementary Figure 4(c) for layer 1, 2 and 3, respectively. An output of 1 or 0 is recorded when the specimen is conductive or nonconductive, respectively.

By utilizing such elementary circuit on the rotational layers of the metamaterial, we develop a method of digital operations to sense the deformation shape of the metamaterial. For instance, at the compact state the system detects a digital output of $Q_1 = 0$, $Q_2 = 0$, and $Q_3 = 0$ that corresponds

to the first buckling mode with a uniform clockwise rotation in all layers. Such investigation illustrates the capability to expand such metamaterial design technique to higher order structures and utilize digital operations for sensing functionalities.

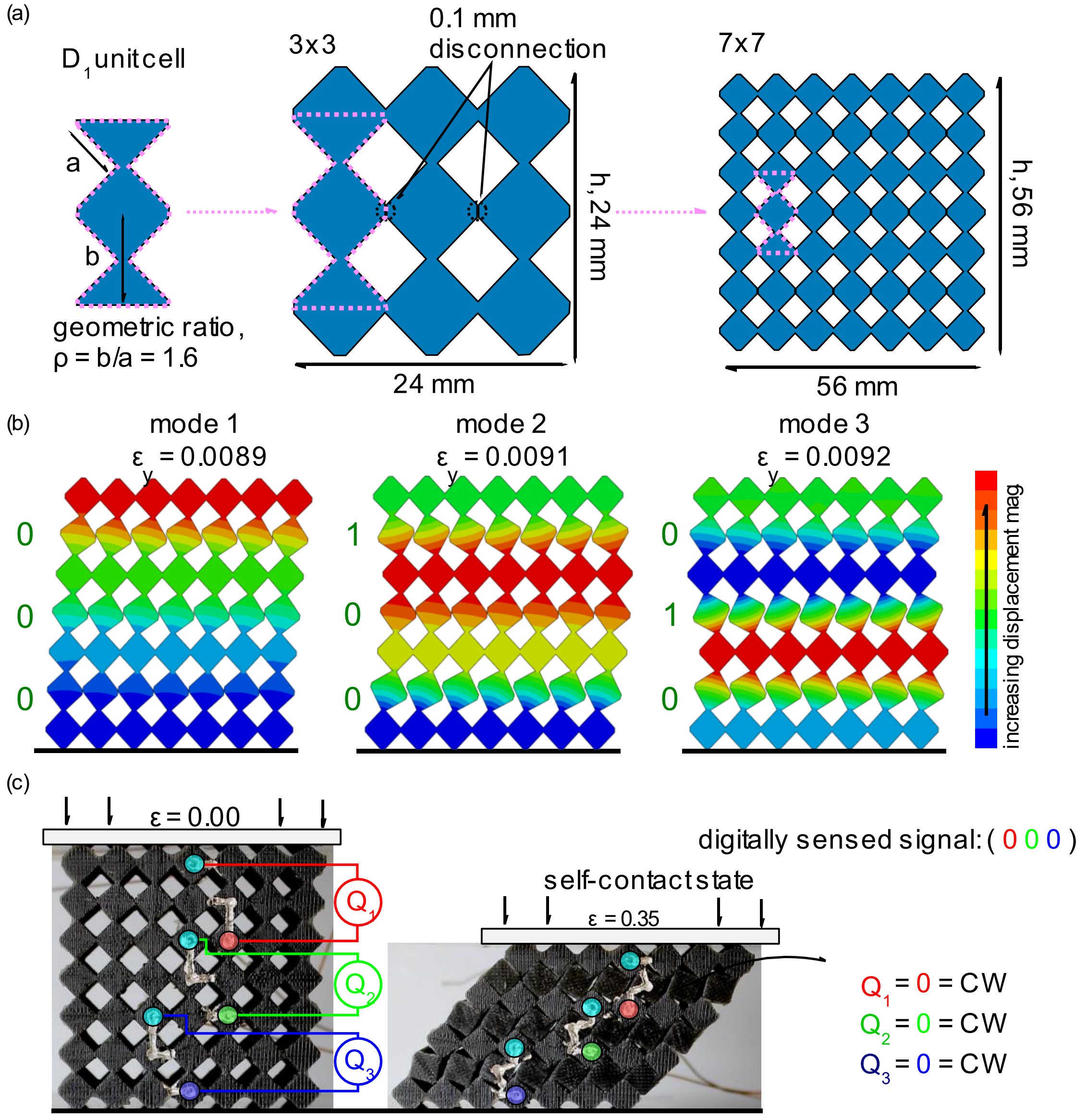

Supplementary Figure 4. (a) Schematic of the D_1 unit cell transformation to a 3x3 metamaterial, and a 7x7 metamaterial. (b) ABAQUS modal analysis results of the 7x7 metamaterial. (c) Experimental

images of the 7x7 metamaterial with Ag-TPU circuit design to detect the deformation states. Depicted in the image is the first buckling mode with a digital output of $Q_1 = 0$, $Q_2 = 0$, and $Q_3 = 0$.

6. Logic gate assembly

The Buffer logic gate Figure 3(d) is the fundamental switch used to formulate all of the metamaterial logic gate. Electrically interfacing the logic gates in series, which is commonly performed in integrated circuits, necessitates a unique approach given the mechanical mode of governing the digital inputs. In this section we discuss three logic gate assembly methods that may be employed for integrated circuit synthesis. Each method is demonstrated using four pairs of logic gate combinations: OR-NAND, NOR-AND, OR-AND, and AND-NAND. These combinations are chosen based on the fundamental Ag-TPU network utilized in each of the gates shown in Figure 4. For instance, the OR gate Figure 4(e) and the NAND gate Figure 4(d) each have two Buffer and/or NOT gates electrically interfaced in parallel. Thus, we combine the gates as OR-NAND to study the outputs of two parallel Ag-TPU networks. The NOR-AND, OR-AND, and AND-NAND are likewise examined below.

6.1 Mechanically coupled inputs

One method of soft, conductive mechanical metamaterial logic gate assembly involves mechanical coupling of the digitized inputs. For instance, Supplementary Figure 5(a) illustrates such method of assembly on an OR and NAND gate by connecting the two metamaterials mechanically in parallel. The result in the continuous metamaterial shown in Supplementary Figure 5(b) that is twice the width dimension of each gate contributor. By serially connecting the electrical output of the OR gate Q_{OR} to the source V_{cc} of the NAND gate, we develop a two-input logic computation with a combined electrical output $Q_{OR\text{-}NAND.}$ Interestingly, the electrical output of this particular

logic gate combination is identical to the output of the XOR gate Q_{XOR}. In fact, the embodiment of OR-NAND in Supplementary Figure 5(b) may be reduced to the XOR gate shown in Supplementary Figure 5(c). This example shows that by increasing the number of columns that are mechanically stressed in parallel, there are not fundamental changes to the logic gate functionality, which is an important factor in light of scalability of the metamaterial network architecture.

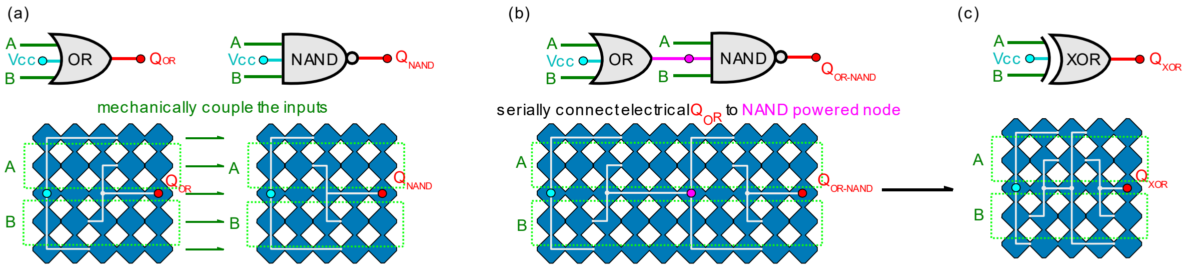

Supplementary Figure 5. (a) Schematic illustrating the method of mechanically coupling the OR and NAND gate. (b) The OR-NAND logic gate combination through mechanical input coupling. (c) Resulting XOR gate for this OR-NAND combination.

The schematic in Supplementary Figure 5(b) suggests that mechanical input coupling bears no direct similarity to conventional logic gate networking. As such, there is no way to validate such digital outputs to equivalent, conventional gate combinations. Nevertheless, here we study this gate assembly method for the NOR-AND, OR-AND, and AND-NAND logic gate combinations to characterize the digital output trends. By mechanically coupling the rotational inputs of the NOR and AND gates Supplementary Figure 6(a), a null digital $Q_{NOR\text{-}AND}$ is output. Similar null digital outputs are the result of the AND and NAND combination in Supplementary Figure 6(c). This is an intrinsic limitation of the serial electrical connection approach when the mechanical inputs are coupled. In other words, if the initial logic gate has a digital output of 0, the computational logic

of the second gate is lost due to the absence of the V_{cc} to the second gate. A second intrinsic limitation to mechanically coupled soft, metamaterial logic gates is observed in the OR-AND combination Supplementary Figure 6(b). For instance, the digital output of the OR-AND $Q_{OR\text{-}AND}$ is identical to that of the AND gate Q_{AND} and thus the output of OR gate is lost. While these are interesting trends for mechanically coupled metamaterial logic gates, the lack of direct electrical gate analogies limits the utility of this metamaterial logic gate assembly method.

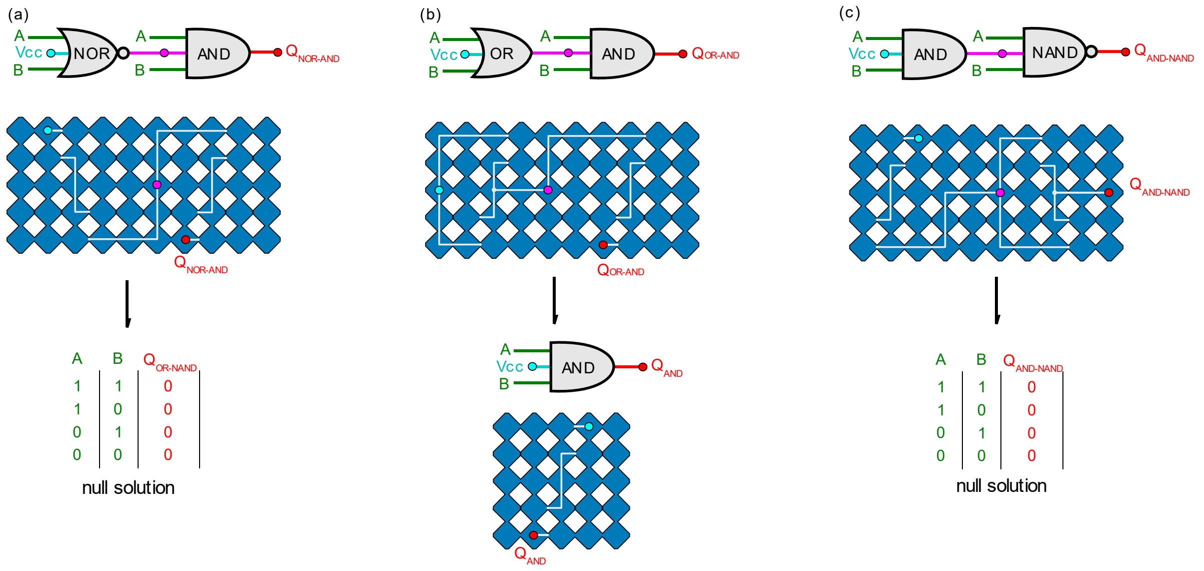

Supplementary Figure 6. The (a) NOR-AND, (b) OR-AND and (c) AND-NAND logic gate combinations and the resultant truth table or equivalent logic gate.

6.2 Mechanically decoupled inputs

In this method we decouple the mechanical inputs and serially interface the Ag-TPU networks from each of two logic gates. As exemplified in Supplementary Figure 7(a) for the OR-NAND combination, the electrical output Q_{OR} drives the input V_{cc} of the NAND gate, while each metamaterial embodiment is structurally separated. This produces 4 mechanical inputs: A and B

into the OR gate, and C and D into the NAND gate. In fact, this approach is identical to an alternative serial mechanical interface that exploits higher order buckling modes of a continuous metamaterial, Supplementary Figure 7(b). The resultant metamaterial logic gate is shown in Supplementary Figure 7(c). This gate leverages 16 (2^4) distinct buckling modes, leading to the $Q_{OR\text{-}NAND}$ truth table in Supplementary Figure 7(d). The digital outputs $Q_{OR\text{-}NAND}$ illustrate a nonconventional logic process without an equivalent electrical gate combination.

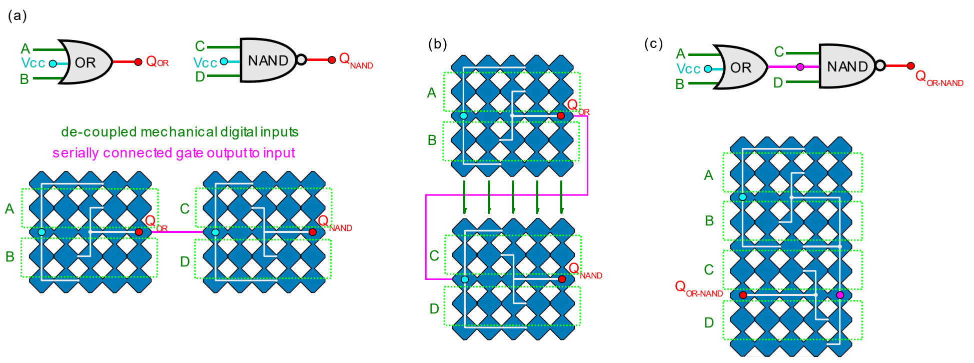

(d)

A	B	C	D	$Q_{OR\text{-}NAND}$
0	0	0	0	0
0	0	1	1	0
0	0	1	0	0
0	0	0	1	0
1	1	0	0	1
1	1	1	1	0
1	1	1	0	1
1	1	0	1	1
1	0	0	0	1
1	0	1	1	0
1	0	1	0	1
1	0	0	1	1
0	1	0	0	1
0	1	1	1	0
0	1	1	0	1
0	1	0	1	1

Supplementary Figure 7. Schematic illustrating the method of mechanically decoupled assembly of the OR and NAND gate through (a) mechanically separation or (b) serial mechanical continuity. (c) The OR-NAND logic gate with four mechanical inputs, A, B, C and D. (d) Resultant digital output truth table.

We examine the NOR-AND, OR-AND, and AND-NAND logic gate combinations using the mechanical decoupling assembly method in Supplementary Figure 8. Each of the gate combinations yields truth table outputs that have no equivalent electrical gate combination due to the fact that the powered V_{cc} of the second gate is governed by the output of the first gate in the electrical network. As a result, it is not possible to validate the truth tables in Supplementary Figure

8(d). On the other hand, this method leads to non-null digital outputs for all logic gate combinations, unlike the mechanical coupling approach evaluated in Sec. 6.3.

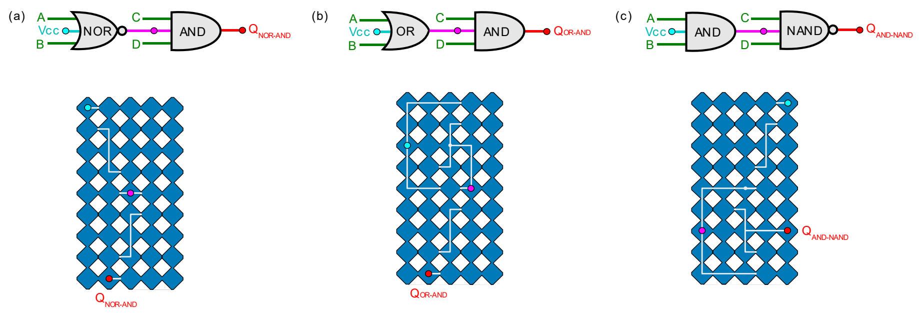

(d)

A	B	C	D	$Q_{NOR-AND}$	Q_{OR-AND}	$Q_{AND-NAND}$
0	0	0	0	0	0	0
0	0	1	1	1	0	0
0	0	1	0	0	0	0
0	0	0	1	0	0	0
1	1	0	0	0	0	1
1	1	1	1	0	1	0
1	1	1	0	0	0	1
1	1	0	1	0	0	1
1	0	0	0	0	0	0
1	0	1	1	0	1	0
1	0	1	0	0	0	0
1	0	0	1	0	0	0
0	1	0	0	0	0	0
0	1	1	1	0	1	0
0	1	1	0	0	0	0
0	1	0	1	0	0	0

Supplementary Figure 8. The (a) NOR-AND, (b) OR-AND and (c) AND-NAND logic gate combinations with (d) respective digital output truth tables.

6.3 Mechanical-electrical network synthesis

Here, we study a final method of assembling the soft, conductive mechanical metamaterial logic gates that synthesizes the gate combinations using the principle of serial and parallel electrical networking of the Buffer and NOT logic gate switches. In this way, exact electrical analogues of the resulting metamaterial logic gate combinations are realized.

As shown in Supplementary Figure 9(a), we transform a two-layer metamaterial into a three-layer metamaterial whereby the networking from the two top-most layers A and B are strategically networked to the bottom-most layer C, Supplementary Figure 9(b). A conventional 3 input OR-NAND gate combination is realized by simultaneously considering that both the OR gate Figure 4(e) and the NAND gate Figure 4(d) have two Buffer and/or NOT gates electrically interfaced in parallel. By synthesizing this fact on the basis of the electrical network, we realize the OR-NAND logic gate combination in Supplementary Figure 9(b) that yields a truth table Supplementary

Figure 9(c) in agreement with the electrical analogue. Since there are 3 rotating layers, the metamaterial exhibits 8 (2^3) buckling modes that correspond to the digital input combinations permitted by an electrical $Q_{OR\text{-}NAND}$ logic gate combination.

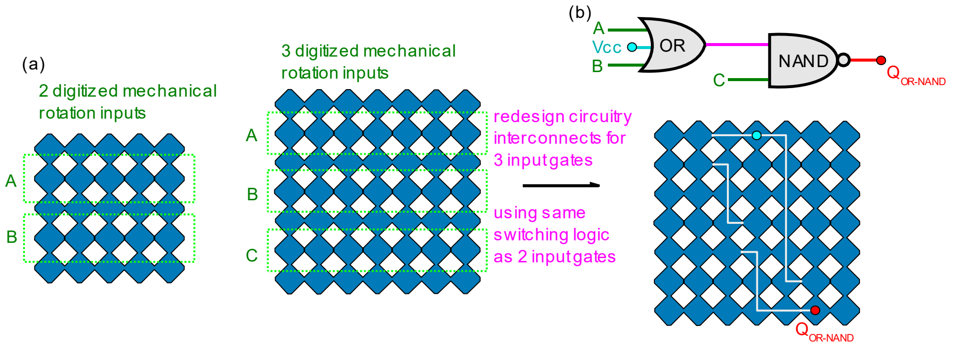

(c)

A	B	C	$Q_{OR\text{-}NAND}$
1	1	1	0
1	1	0	1
1	0	0	1
0	0	0	1
0	1	1	0
0	0	1	1
1	0	1	0
0	1	0	1

Supplementary Figure 9. (a) Schematic illustrating a mechanical-electrical network synthesis approach of metamaterial logic gate combination. (b) The OR-NAND logic gate combination with three mechanical inputs A, B, and C. (c) Resultant digital output truth table.

We use this strategic logic gate synthesis method to formulate the NOR-AND, OR-AND, and AND-NAND gate combinations in Figure S10. For instance, the NOR-AND Supplementary Figure 10(a) utilizes the NOR circuitry Figure 4(f) on input A and B in series with a single switch (Buffer gate) via mechanical rotation input C. Similar approaches to electrically network the OR gate with the AND gate are employed in Supplementary Figure 10(b) and to network an AND gate with a NAND gate in Supplementary Figure 10(c). The truth tables for these gates in Supplementary Figure 10(d) agree with each of the conventional electrical analogues. Thus, by a synthesis of metamaterial mechanical layering, Ag-TPU networking, and electrical interfaces that accommodate the necessary serial or parallel digital input needs identified from the constituent logic gates in Figure 4 of the main text, one may realize any arbitrary combination of the soft, conductive mechanical metamaterial logic gates.

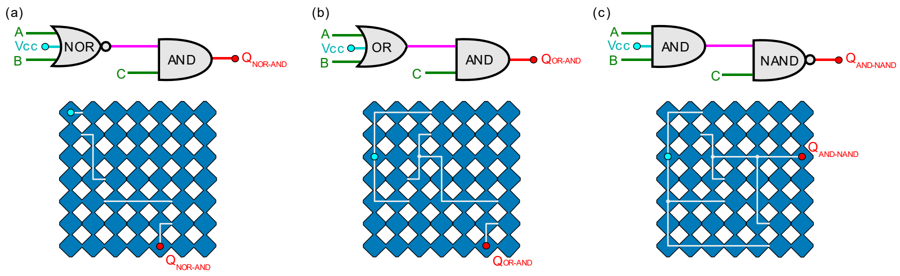

(d)

A	B	C	$Q_{NOR-AND}$	Q_{OR-AND}	$Q_{AND-NAND}$
1	1	1	0	1	0
1	1	0	0	0	1
1	0	0	0	0	1
0	0	0	0	0	1
0	1	1	0	1	1
0	0	1	1	0	1
1	0	1	0	1	1
0	1	0	0	0	1

Supplementary Figure 10. The (a) NOR-AND, (b) OR-AND and (c) AND-NAND logic gate combinations and (d) respective digital output truth tables.
